# Unveiling isoleucyl‐tRNA synthetase 2 as a novel driver of breast cancer via β‐catenin pathway activation

**DOI:** 10.1002/ccs3.70094

**Published:** 2026-07-12

**Authors:** Xi Yang, Ya Wang, Yanjiao Yi, Yang Yang, Hongjiang Wang

**Affiliations:** ^1^ Department of Breast Surgery The First Affiliated Hospital of Dalian Medical University Dalian China; ^2^ Department of Plastic Surgery The First Affiliated Hospital of Dalian Medical University Dalian China

**Keywords:** β‐catenin, breast cancer, IARS2, malignant progression, ubiquitination

## Abstract

Breast cancer represents the most common malignant tumor in women globally. Isoleucyl‐tRNA synthetase 2 (IARS2), a member of the aminoacyl‐tRNA synthetase family, promotes tumorigenesis across various cancers. Herein, the effects and mechanisms of IARS2 in breast cancer were investigated. IARS2 is upregulated in breast cancer tissues compared to normal tissues, correlating with the poor prognosis of patients with breast cancer. In order to knock down and overexpress IARS2, plasmids carrying short hairpin RNA targeting IARS2 and IARS2‐overexpression plasmids were transfected into breast cancer cells, respectively. Our results demonstrated that silencing IARS2 repressed breast cancer cell progression. IARS2 deficiency attenuated tumor growth in MDA‐MB‐231 cell xenograft mouse models. Conversely, overexpression of IARS2 aggravated malignant cell behaviors in vitro and accelerated tumor growth in vivo. Mechanistically, IARS2 depletion stimulated ubiquitination‐mediated degradation of β‐catenin in breast cancer cells. Notably, the β‐catenin inhibitor XAV‐939 reversed IARS2‐driven breast cancer malignant progression. Furthermore, differentially expressed genes were identified in IARS2‐overexpressed breast cancer cells with or without XAV‐939 in the light of mRNA‐seq results. Overall, our findings uncovered that silencing IARS2 blocked the β‐catenin signaling axis, thereby impeding breast cancer progression. IARS2 might become a promising novel therapeutic target for breast cancer intervention.

## Graphical abstract

Isoleucyl‐tRNA synthetase 2 (IARS2) stabilizes β‐catenin by reducing its phosphorylation at Ser33/37/Thr41 and ubiquitin‐proteasome degradation, thereby promoting β‐catenin nuclear accumulation and transcriptional activation of downstream targets, including inhibitor of DNA binding 3, lactate dehydrogenase A, and Drosha ribonuclease III. This axis drives breast cancer cell proliferation while suppressing apoptosis, highlighting IARS2 as a critical regulator of β‐catenin signaling in breast cancer progression.

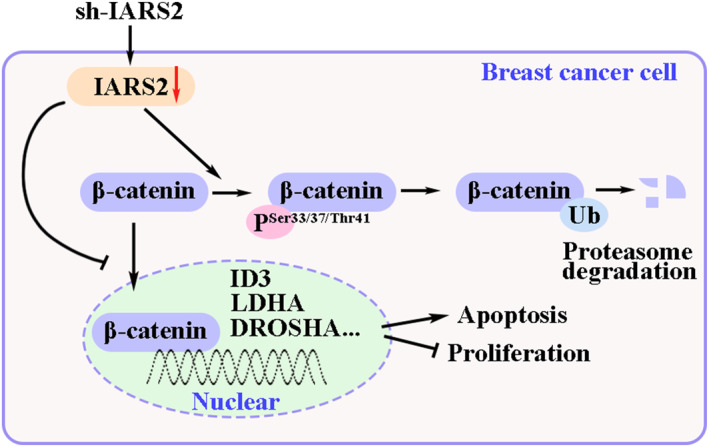

## 1 INTRODUCTION

Breast cancer represents the leading cause of cancer mortality among women worldwide.^1^ Breast cancer is a heterogeneous disease, classified into three major subtypes according to the pattern of expression of estrogen receptor, progesterone receptor, and human epidermal growth factor receptor 2 (HER2): luminal A/B (HR positive), HER2‐positive, and triple‐negative breast cancer.^2^ Current therapeutic strategies for breast cancer predominantly rely on surgery, chemotherapy, and targeted therapy.^3^, ^4^ Despite significant advancements, many patients develop drug resistance or disease recurrence after treatment. Therefore, the identification of novel therapeutic targets holds promise for improving the treatment outcomes of patients with breast cancer.

Aminoacyl‐tRNA synthetases (ARSs) are capable of catalyzing the binding of specific amino acids to their corresponding tRNAs, playing an essential role in protein synthesis.^5^ ARSs participate in various physiological and pathological processes, especially the occurrence and development of cancer, by regulating cell growth, differentiation, cytokine activity, RNA splicing, and angiogenesis.^6^

Isoleucyl‐tRNA synthetase 2 (IARS2), also known as IleRS, belongs to the ARS family. The IARS2 gene encodes mitochondrial isoleucine tRNA synthetase.^7^ IARS2 has been reported as an oncogene in various cancers, including glioblastoma,^8^ non‐small‐cell lung cancer,^9^, ^10^ gastric cancer,^11^ colon cancer,^12^, ^13^ and cervical cancer.^14^ For instance, IARS2 expression is higher in short‐term survivors of glioblastoma than in long‐term survivors, suggesting that high IARS2 expression is a risk factor for glioblastoma.^8^ Knockdown of IARS2 inhibits non‐small‐cell lung cancer progression,^9^ in which the AKT/mTOR pathway exerts an important role.^10^ IARS2 downregulation suppresses gastric cancer cell growth by regulating the phosphorylation of cell cycle‐related proteins.^11^ IARS2 promotes colon cancer cell proliferation, inhibits apoptosis, and enhances tumorigenicity.^12^ In addition, loss of IARS2 impairs mitochondrial function and suppresses malignant phenotypes in cervical cancer cells.^14^ Knocking down IARS2 restrains proliferation in human osteosarcoma cells,^15^ melanoma cells,^16^ and acute myeloid leukemia cells.^17^ These studies indicate that IARS2 plays a significant role in tumor development. Interestingly, IARS2 expression is upregulated in breast cancer, and the high expression predicts poor prognosis on the basis of the public databases, such as UALCAN‐TCGA and Kaplan–Meier plotter. Taken together, we speculated that IARS2 might be involved in the progression of breast cancer.

Importantly, a study has shown that IARS2 deficiency in human umbilical vein endothelial cells reduces the expression of β‐catenin.^18^ Furthermore, existing research has demonstrated that IARS2 promotes proliferation and metastasis by stabilizing β‐catenin in pancreatic ductal adenocarcinoma.^19^ Accumulating studies have also reported that aberrant activation of β‐catenin is closely associated with malignant progression in breast cancer. For instance, long‐term exposure to 4‐hydroxybenzophenone has been shown to upregulate β‐catenin expression and enhance its protein stability, thereby accelerating breast cancer metastasis.^20^ FXYD domain‐containing ion transport regulator 1 can reduce β‐catenin stability and impair its nuclear translocation, leading to the repression of breast cancer metastasis.^21^ Interestingly, there is evidence indicating that members of the ARS family are linked to β‐catenin regulation. Glutamyl‐prolyl‐tRNA synthetase, a member of the ARS family, directly binds to SCY1‐like protein 2, activates the Wnt/GSK‐3β/β‐catenin signaling pathway, and boosts the nuclear accumulation of β‐catenin, thereby advancing gastric cancer cell proliferation and tumor growth.^22^ Another ARS family member, seryl‐tRNA synthetase, suppresses β‐catenin expression by inhibiting Wnt signaling, consequently restraining breast cancer cell growth and metastasis.^23^ Based on these findings, we hypothesized that IARS2 may influence breast cancer progression through the regulation of β‐catenin.

In this study, we investigated whether IARS2 affects the development of breast cancer and whether β‐catenin signaling is involved. Moreover, we explored whether IARS2 regulates the transcription of β‐catenin downstream genes in breast cancer cells in combination with transcriptome sequencing. Taken together, IARS2 might serve as a potential therapeutic target for breast cancer treatment.

## 2 MATERIALS AND METHODS

### 2.1 Clinical samples

A total of 30 human breast cancer tissues and corresponding para‐carcinoma tissues were acquired for the verification of IARS2 mRNA expression. In addition, we collected 56 breast cancer tissues for IARS2 protein level detection by immunohistochemistry (IHC). Informed consent was obtained from each patient before sample collection.

### 2.2 Cell culture

Human breast cancer cell lines (MDA‐MB‐231 and BT‐20 cells) were purchased from Cellverse Co., Ltd. in November 2024. The details of cell culture are provided in Supporting Information S1.

### 2.3 Cell transfection

MDA‐MB‐231 and BT‐20 cells were transfected with plasmids carrying short hairpin RNA targeting IARS2 (sh‐IARS2) and IARS2‐overexpression plasmids (Ov‐IARS2) using Lipofectamine 3000 (Invitrogen). The nontransfected cells and the cells transfected with negative control shRNA (sh‐NC) and empty vectors (vector) were used as the control. The target sequence for sh‐IARS2 was 5′‐GTACTTGCAGTCATCCATTAA‐3′. After transfection, stable clones were selected. Five sh‐IARS2 clones (sh‐IARS2‐1, sh‐IARS2‐2, sh‐IARS2‐3, sh‐IARS2‐4, and sh‐IARS2‐5) and five Ov‐IARS2 clones (Ov‐IARS2‐1, Ov‐IARS2‐2, Ov‐IARS2‐3, Ov‐IARS2‐4, and Ov‐IARS2‐5) were screened by qRT‐PCR, and the two clones with the most efficient reduction (sh‐IARS2‐3 and sh‐IARS2‐4) and the two clones with the highest IARS2 mRNA expression (Ov‐IARS2‐2 and Ov‐IARS2‐5) were selected for Western blot validation and subsequent functional experiments.

### 2.4 Xenograft mouse model

Female BALB/c nude mice were purchased from Liaoning Changsheng Biotechnology Co., Ltd. The establishment of a xenograft mouse model was performed according to previous literature.^24^ The details are provided in Supporting Information S1. This study was approved by the Experimental Animal Ethical Committee of Dalian Medical University (AEE24319).

### 2.5 Immunohistochemistry

The details are provided in Supporting Information S1.

### 2.6 Cell treatment

The MDA‐MB‐231 cells transfected with sh‐IARS2‐3 and sh‐NC were treated with cycloheximide or MG132, referring to previous literature.^19^ The details are provided in Supporting Information S1.

### 2.7 Cell proliferation

Cell Counting Kit‐8 (CCK‐8) was applied to test cell viability. The details are provided in Supporting Information S1.

### 2.8 Cell cycle and apoptosis

The detection kits of cell cycle and apoptosis were purchased from KeyGEN Biotechnology Co., Ltd. The details are provided in Supporting Information S1.

### 2.9 Cell migration

The details are provided in Supporting Information S1.

### 2.10 Cell invasion

The details are provided in Supporting Information S1.

### 2.11 Immunofluorescence

The details are provided in Supporting Information S1.

### 2.12 Dual‐luciferase reporter assay

The details are provided in Supporting Information S1.

### 2.13 mRNA‐seq

Total RNA was extracted from MDA‐MB‐231 cells transfected with vector and from the cells transfected with Ov‐IARS2‐2 treated with dimethyl sulfoxide (DMSO) and from the IARS2‐2‐overexpressed cells treated with XAV‐939. The samples were entrusted to Novogene for transcriptome sequencing on Illumina platforms. Differentially expressed genes (DEGs) were identified according to the criteria of |log_2_ fold change| > 0.8 and *p* < 0.05.

### 2.14 Real‐time PCR

The details are provided in Supporting Information S1.

### 2.15 Co‐immunoprecipitation

The Pierce Co‐Immunoprecipitation Kit (Thermo Scientific) was applied to measure the ubiquitination of β‐catenin in transfected breast cancer cells treated with MG132. The details are provided in Supporting Information S1.

### 2.16 Western blot

The details are provided in Supporting Information S1.

### 2.17 Statistical analysis

Data are presented as the mean ± standard deviation and were analyzed using GraphPad Prism software. Statistical differences between two groups were assessed by a *t*‐test. Statistical differences among more than two groups were determined by ordinary one‐way ANOVA. Values of *p* < 0.05 were considered significantly different.

## 3 RESULTS

### 3.1 Upregulated IARS2 in breast cancer was associated with poor prognosis

**FIGURE 1:**
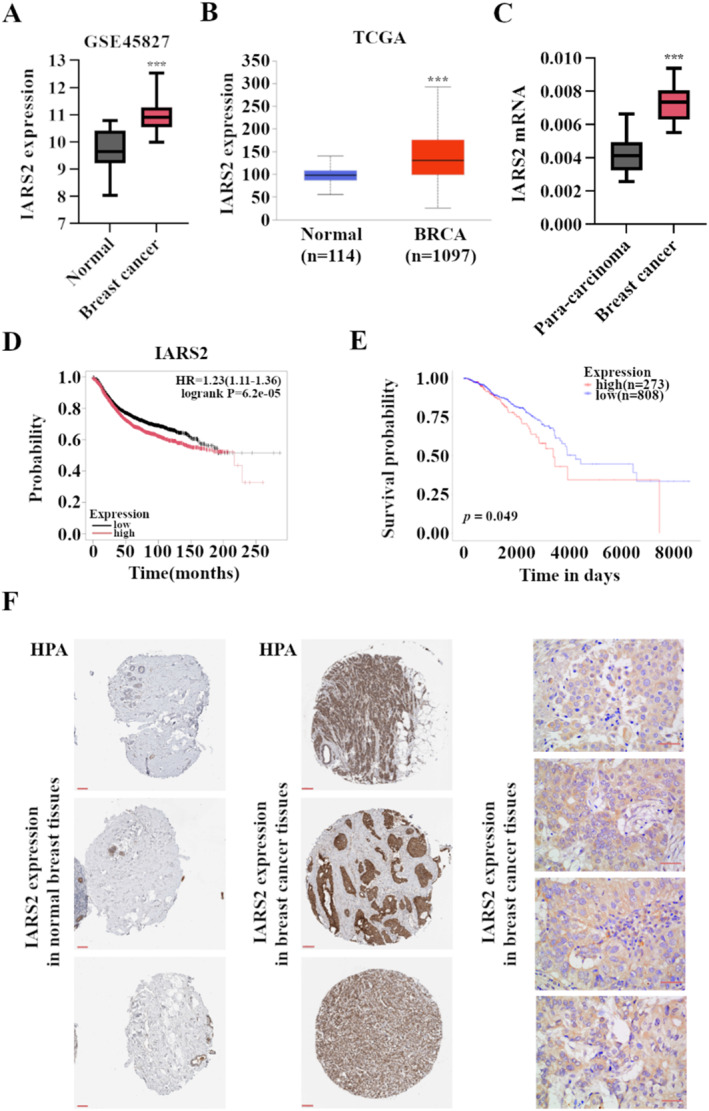
IARS2 was upregulated in human breast cancer tissues and correlated with the survival of patients with breast cancer. (A) Upregulated IARS2 expression in 130 primary invasive breast cancer tissues compared with 11 normal tissues based on GSE45827. (B) The expression of IARS2 in BRCA on the basis of the TCGA database from the UALCAN website (https://ualcan.path.uab.edu/index.html). (C) Real‐time PCR determined the mRNA expression of IARS2 in 30 pairs of breast cancer tissues and adjacent normal tissues. (D) Kaplan–Meier survival curve of the high‐ and low‐IARS2‐expression groups in breast cancer according to gene chip data from the Kaplan–Meier plotter database (https://kmplot.com/analysis/). (E) The survival curve of IARS2 in the TCGA database from the UALCAN website showed that high expression of IARS2 was associated with poor prognosis in BRCA. (F) IARS2 protein expression in normal breast tissues and breast cancer tissues was presented based on the HPA database. The scale bar is 100 μm. Representative IHC images of IARS2 staining in 56 breast cancer tissues. The scale bar is 50 μm. ***p < 0.001. BRCA, breast invasive carcinoma; HPA, Human Protein Atlas; IARS2, isoleucyl‐tRNA synthetase 2; IHC, immunohistochemistry.

GSE45827 contains 130 primary invasive breast cancer tissues and 11 normal tissues. By retrieving the data from GSE45827, it was found that IARS2 was upregulated in primary invasive breast cancer tissues compared with normal tissues (Figure 1A). Besides, upregulated IARS2 in breast invasive carcinoma (BRCA) was displayed in the light of the TCGA database from the UALCAN website (Figure 1B). We investigated that IARS2 mRNA expression in breast cancer tissues was higher than that in adjacent normal tissues (Figure 1C). The Kaplan–Meier plotter database and the TCGA database showed that high expression of IARS2 indicated a poor prognosis of patients with breast cancer (Figure 1D,E). The IHC staining of IARS2 in human breast cancer tissues was presented. Additionally, the levels of IARS2 protein expression in normal breast tissues and breast cancer tissues were exhibited using data from the Human Protein Atlas database (Figure 1F). These results confirmed the upregulated expression of IARS2 in breast cancer, and there was a correlation between high IARS2 expression and the poor prognosis of breast cancer.

### 3.2 IARS2 enhanced breast cancer cell proliferation and accelerated cell cycle progression

**FIGURE 2:**
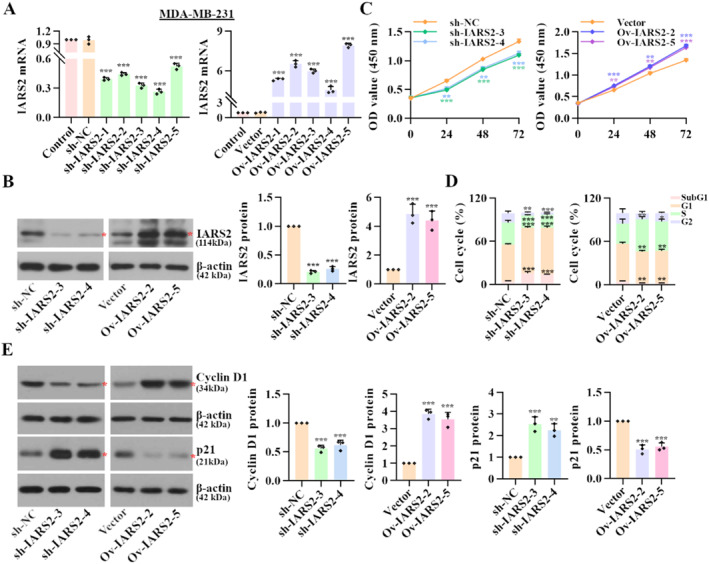
IARS2 accelerated cell proliferation and cycle progression in MDA‐MB‐231 cells. Plasmids carrying sh‐IARS2 and Ov‐IARS2 were transfected into MDA‐MB‐231 cells. (A) We screened five monoclonal stable cells from each of the sh‐IARS2‐transfected cells and the Ov‐IARS2‐transfected cells for validation of IARS2 mRNA expression using real‐time PCR. Cells transfected with sh‐IARS2‐3 and sh‐IARS2‐4 and cells transfected with Ov‐IARS2‐2 and Ov‐IARS2‐5 displayed better transfection efficiency compared to the other cells. (B) IARS2 protein expression was validated by Western blot in cells transfected with sh‐IARS2‐3 and sh‐IARS2‐4 or Ov‐IARS2‐2 and Ov‐IARS2‐5. (C) Cell proliferation was analyzed by CCK‐8 assays. (D) Cell cycle was assessed by flow cytometry. (E) Western blot analysis of cyclin D1 and p21. *n* = 3 per group. *p < 0.05, **p < 0.01, ***p < 0.001. CCK‐8, Cell Counting Kit‐8; IARS2, isoleucyl‐tRNA synthetase 2; Ov‐IARS2, IARS2‐overexpression plasmids; sh, short hairpin; sh‐IARS2, short harpin RNA targeting IARS2.

**FIGURE 3:**
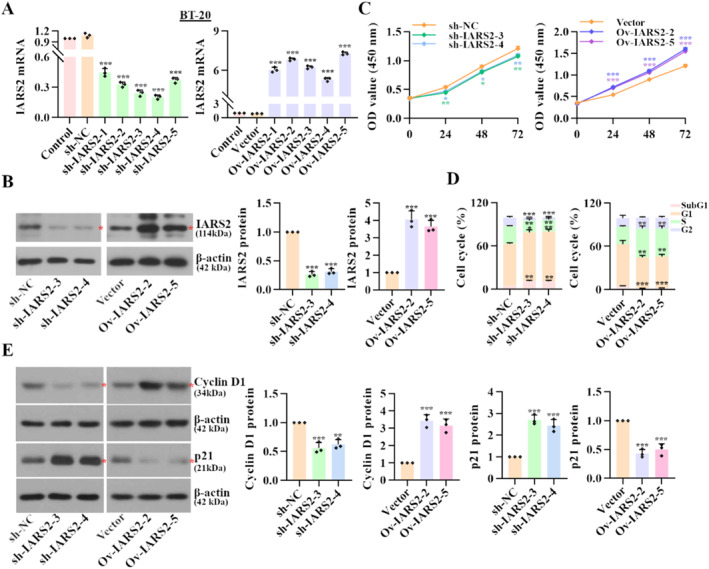
IARS2 stimulated cell proliferation and cycle progression in BT‐20 cells. (A) Validation of IARS2 mRNA expression using real‐time PCR. (B) IARS2 protein expression by Western blot in BT‐20 cells transfected with sh‐IARS2‐3 and sh‐IARS2‐4 or Ov‐IARS2‐2 and Ov‐IARS2‐5. (C) Cell proliferation by CCK‐8 assays. (D) Cell cycle by flow cytometry. (E) Protein examination of cyclin D1 and p21 by Western blot. *n* = 3 per group. *p < 0.05, **p < 0.01, ***p < 0.001. CCK‐8, Cell Counting Kit‐8; IARS2, isoleucyl‐tRNA synthetase 2; Ov‐IARS2, IARS2‐overexpression plasmids; sh‐IARS2, short harpin RNA targeting IARS2.

We examined the effects of IARS2 on the cell proliferation and cycle progression in MDA‐MB‐231 (Figure 2) and BT‐20 cells (Figure 3). Silencing and overexpression plasmids targeting IARS2 were transfected into breast cancer cells, and then monoclonal stable cell lines were generated. Successful transfection was confirmed in five sh‐IARS2 clones and five Ov‐IARS2 clones by real‐time PCR (Figures 2A and 3A). Of the five clones screened at the mRNA level, the two clones with the most pronounced and stable expression changes (sh‐IARS2‐3 and sh‐IARS2‐4; Ov‐IARS2‐2 and Ov‐IARS2‐5) were selected for subsequent functional assays, and IARS2 protein levels were confirmed to be directionally consistent with the mRNA data (Figures 2B and 3B). In addition, silencing IARS2 suppressed breast cancer cell proliferation. To the contrary, overexpressing IARS2 enhanced cell proliferation in breast cancer (Figures 2C and 3C). IARS2 knockdown led to cell cycle arrest, whereas IARS2 overexpression expedited cell cycle progression in breast cancer (Figures 2D and 3D, Supporting Information S1: Figure S1A,B). Consistently, IARS2 knockdown reduced the protein levels of cyclin D1, a positive regulator of G1/S progression, although increasing the levels of p21, a cyclin‐dependent kinase inhibitor that promotes cell cycle arrest (Figures 2E and 3E). These results suggested that IARS2 promoted breast cancer cell proliferation and cycle progression.

### 3.3 IARS2 knockdown accelerated cell apoptosis and inhibited cell migration and invasion in breast cancer cells

**FIGURE 4:**
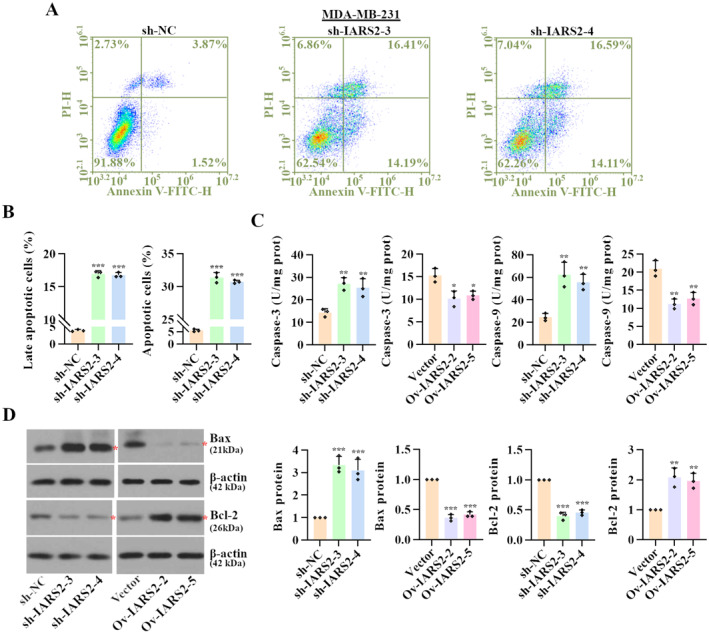
Silencing IARS2 increased MDA‐MB‐231 cell apoptosis. (A) Cell apoptosis was evaluated using flow cytometry in the IARS2‐knockdown MDA‐MB‐231 cells. (B) The rates of late apoptotic cells and apoptotic cells were calculated. (C) Caspase‐3 and caspase‐9 activity. (D) Western blot analysis of Bax and Bcl‐2. *n* = 3 per group. *p < 0.05, **p < 0.01, ***p < 0.001. IARS2, isoleucyl‐tRNA synthetase 2.

**FIGURE 5:**
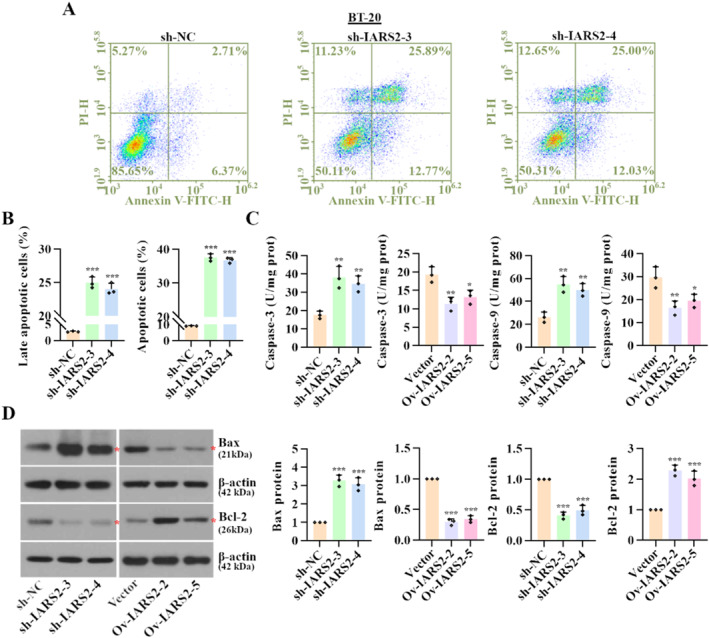
IARS2 reduction promoted BT‐20 cell apoptosis. (A) Cell apoptosis was evaluated using flow cytometry in the IARS2‐knockdown BT‐20 cells. (B) The rates of late apoptotic cells and apoptotic cells. (C) Caspase‐3 and caspase‐9 activity. (D) Western blot analysis of Bax and Bcl‐2. *n* = 3 per group. *p < 0.05, **p < 0.01, ***p < 0.001. IARS2, isoleucyl‐tRNA synthetase 2.

The apoptotic cells were augmented in the IARS2‐silenced MDA‐MB‐231 cells (Figure 4A,B). The reduction of IARS2 increased the activity of apoptosis‐related caspase‐3 and caspase‐9 proteins in the cells (Figure 4C). Conversely, reduced numbers of apoptotic cells and the inhibition of caspase‐3 and caspase‐9 were exhibited in the MDA‐MB‐231 cells treated with Ov‐IARS2‐2 and Ov‐IARS2‐5 (Figure 4C, Supporting Information S1: Figure S2A). Moreover, it was found that IARS2 knockdown increased the proapoptotic Bax protein level and inhibited the antiapoptotic Bcl‐2 protein level, whereas IARS2 overexpression caused the opposite trend (Figure 4D). The same results were also observed in BT‐20 cells (Figure 5A–D, Supporting Information S1: Figure S2B). These results indicated that IARS2 suppressed breast cancer apoptosis.

In addition, wound healing assays were performed to evaluate the effect of IARS2 on breast cancer cell migration. As shown in Supporting Information S1: Figure S3A, IARS2 knockdown reduced cell migration, whereas IARS2 overexpression accelerated wound closure in MDA‐MB‐231 cells. The invasive ability of MDA‐MB‐231 cells was reduced by IARS2 knockdown, whereas IARS2 overexpression led to an increase in cell invasion (Supporting Information S1: Figure S3B). These consistent results were also presented in BT‐20 cells (Supporting Information S1: Figure S4A,B). These data indicated that IARS2 enhanced the migratory capacity and invasive potential of breast cancer cells.

### 3.4 IARS2 facilitated breast cancer growth in the xenograft mouse model, involving β‐catenin signaling

**FIGURE 6:**
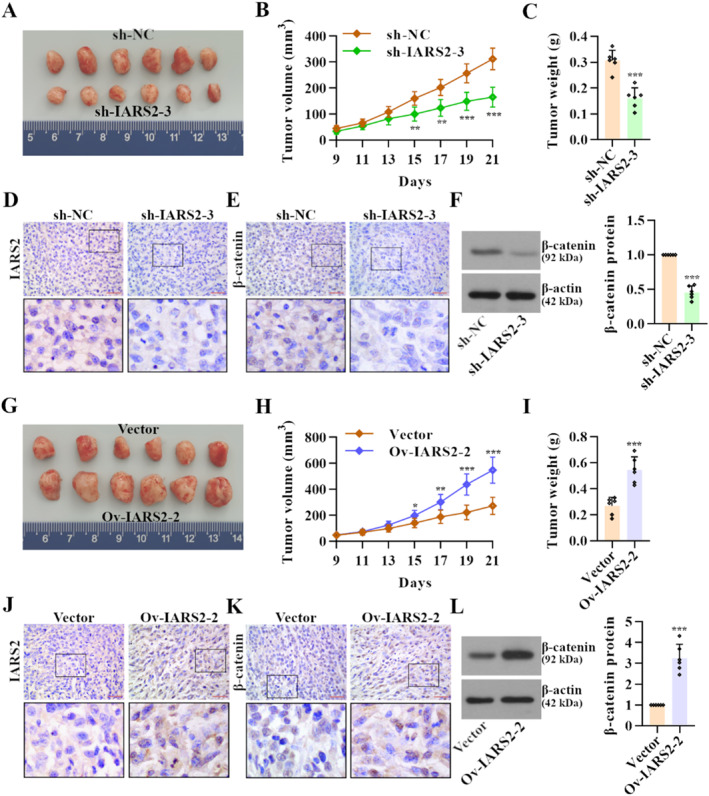
IARS2 promoted tumor growth in the MDA‐MB‐231 breast cancer cell xenograft mouse model, which might involve the regulation of β‐catenin. MDA‐MB‐231 cells transfected with sh‐IARS2‐3 and Ov‐IARS2‐2 were injected subcutaneously into mice. (A–C) The effects of silencing IARS2 on breast cancer tumor volume and weight. (D, E) IHC detected the levels of IARS2 and β‐catenin in the tumor tissues derived from the mice injected with IARS2‐silenced cells. The scale bar is 50 μm. (F) β‐Catenin protein expression in the tumor tissues by Western blot analysis. (G–I) The effects of overexpressing IARS2 on breast cancer tumor volume and weight. (J, K) IHC detected the levels of IARS2 and β‐catenin in the tumor tissues derived from the mice injected with IARS2‐overexpressed cells. The scale bar is 50 μm. (L) Western blot was performed to examine β‐catenin protein expression in the tumor tissues. *n* = 6 per group. *p < 0.05, **p < 0.01, ***p < 0.001. IARS2, isoleucyl‐tRNA synthetase 2; IHC, immunohistochemistry.

To determine the effects of IARS2 on breast cancer tumor growth in vivo, the mice were injected subcutaneously with IARS2‐silenced or IARS2‐overexpressed cells. As shown in Figure 6A–C, IARS2 knockdown relieved the increase in tumor volume and decreased tumor weight in breast cancer. IHC staining showed that the injection of IARS2‐silenced cells inhibited the expression of IARS2 successfully in the mouse tumor tissues (Figure 6D). Notably, IHC showed that silencing IARS2 led to the diminution of β‐catenin levels in the tumor tissues (Figure 6E), which was also validated using Western blot (Figure 6F). Conversely, IARS2 overexpression accelerated breast cancer tumor growth in mice (Figure 6G–I). IARS2 expression was increased in the tumor tissues of the mice injected with IARS2‐overexpressed cells (Figure 6J). As expected, overexpressing IARS2 upregulated β‐catenin protein levels in the tumor tissues (Figure 6K,L). Besides, IARS2 silencing inhibited the levels of β‐catenin targets c‐Myc, cyclin D1, and Axin‐2 (Supporting Information S1: Figure S5A), whereas IARS2 overexpression enhanced the expression of these target genes (Supporting Information S1: Figure S5B). These results suggested that β‐catenin signaling was activated by the tumor‐promoting factor IARS2 in breast cancer.

### 3.5 Silencing IARS2 promoted the ubiquitination and degradation of β‐catenin in breast cancer cells

**FIGURE 7:**
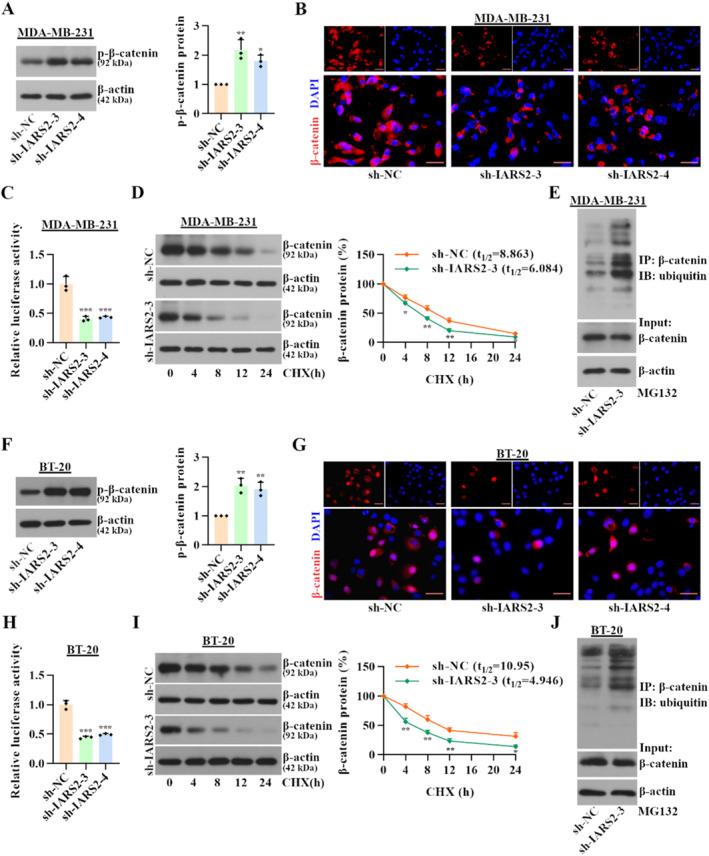
IARS2 knockdown suppressed β‐catenin signaling in breast cancer cells. (A, F) p‐β‐Catenin (Ser33/37/Thr41) protein expression was enhanced in MDA‐MB‐231 and BT‐20 cells with sh‐IARS2‐3 and sh‐IARS2‐4 compared with those with sh‐NC. (B, G) Immunofluorescence staining was performed to observe β‐catenin nuclear localization in IARS2‐silenced cells. Scale bar is 50 μm. (C, H) The cells were cotransfected with the β‐catenin‐mediated transcriptional reporter TOPflash and pRL‐TK plasmids to conduct a dual‐luciferase reporter assay. The relative luciferase activity (firefly/Renilla) was calculated to reflect the relative β‐catenin activity. (D, I) The cells were treated with CHX and measured for β‐catenin protein expression after processing for 0, 4, 8, 12, and 24 h. IARS2 knockdown expedited β‐catenin degradation. (E, J) The proteasome inhibitor MG132 was used to treat the transfected cells, and then co‐IP was performed to test β‐catenin ubiquitination. *n* = 3 per group. *p < 0.05, **p < 0.01, ***p < 0.001. CHX, cycloheximide; co‐IP, co‐immunoprecipitation; IARS2, isoleucyl‐tRNA synthetase 2.

It is universally known that β‐catenin is closely related to the occurrence and development of various cancers and is recognized as a tumor driver.^25^ A previous study has reported that IARS2 knockdown is capable of suppressing the expression of β‐catenin in human umbilical vein endothelial cells.^18^ Herein, silencing IARS2 elevated the expression of p‐β‐catenin (Figure 7A). The β‐catenin levels in the nucleus were decreased in the IARS2 knockdown MDA‐MB‐231 cell nuclei (Figure 7B). The dual‐luciferase reporter assay suggested that β‐catenin activity was inhibited by IARS2 knockdown in MDA‐MB‐231 cells (Figure 7C). Furthermore, it was found that the lack of IARS2 accelerated CHX‐induced degradation of β‐catenin protein (Figure 7D). Meanwhile, β‐catenin ubiquitination was enhanced by IARS2 knockdown (Figure 7E). Besides, these results were also presented in BT‐20 cells (Figure 7F–J). These findings indicated that silencing IARS2 contributed to the suppression of β‐catenin signaling in breast cancer cells.

### 3.6 β‐Catenin inhibition blocked IARS2‐triggered malignant progression in breast cancer cells

**FIGURE 8:**
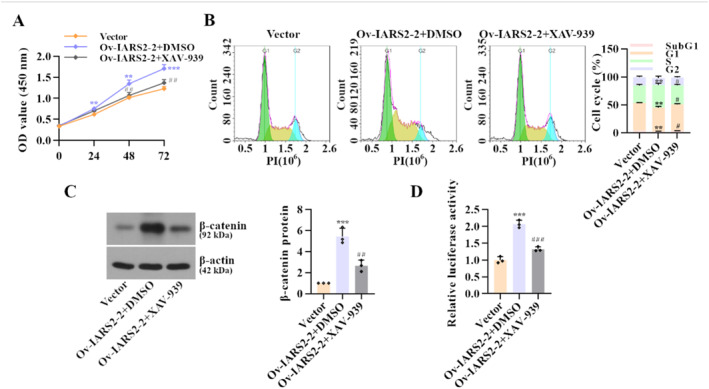
β‐Catenin was required for IARS2 to facilitate breast cancer cell progression. (A) CCK‐8 was used to examine cell proliferation in Ov‐IARS2‐2‐transfected MDA‐MB‐231 cells treated with the β‐catenin inhibitor XAV‐939. (B) Cell cycle progression was assessed in the cells via flow cytometry. (C) β‐Catenin protein levels were detected using Western blot. (D) Relative β‐catenin activity was determined from the relative luciferase activity (firefly/Renilla) using a dual‐luciferase reporter assay. *n* = 3 per group. **p < 0.01, ***p < 0.001 versus the vector group. ^#^ *p* < 0.05, ^##^ *p* < 0.01, ^###^ *p* < 0.001 versus the Ov‐IARS2‐2 + DMSO group. CCK‐8, Cell Counting Kit‐8; DMSO, dimethyl sulfoxide; Ov‐IARS2, isoleucyl‐tRNA synthetase 2‐overexpression plasmids.

To further investigate the relationship of the effects of IARS2 on breast cancer and β‐catenin signaling, the β‐catenin inhibitor XAV‐939 was used to treat IARS2‐overexpressed MDA‐MB‐231 cells. The IARS2 overexpression induced an increase in cell proliferation that was diminished by XAV‐939 in MDA‐MB‐231 cells (Figure 8A). Cell cycle progression stimulated by IARS2 was reversed by XAV‐939 in the cells (Figure 8B). β‐Catenin protein expression was upregulated in the Ov‐IARS2‐transfected cells, whereas the expression was reduced after XAV‐939 treatment (Figure 8C). Notably, the dual‐luciferase reporter assay indicated that IARS2‐promoted β‐catenin activity was repressed by XAV‐939 in the cells (Figure 8D). These data demonstrated that IARS2 might stimulate breast cancer cell proliferation and cycle progression via the β‐catenin pathway.

### 3.7 mRNA‐seq analyzed β‐catenin‐related genes regulated by IARS2 in breast cancer cells

**FIGURE 9:**
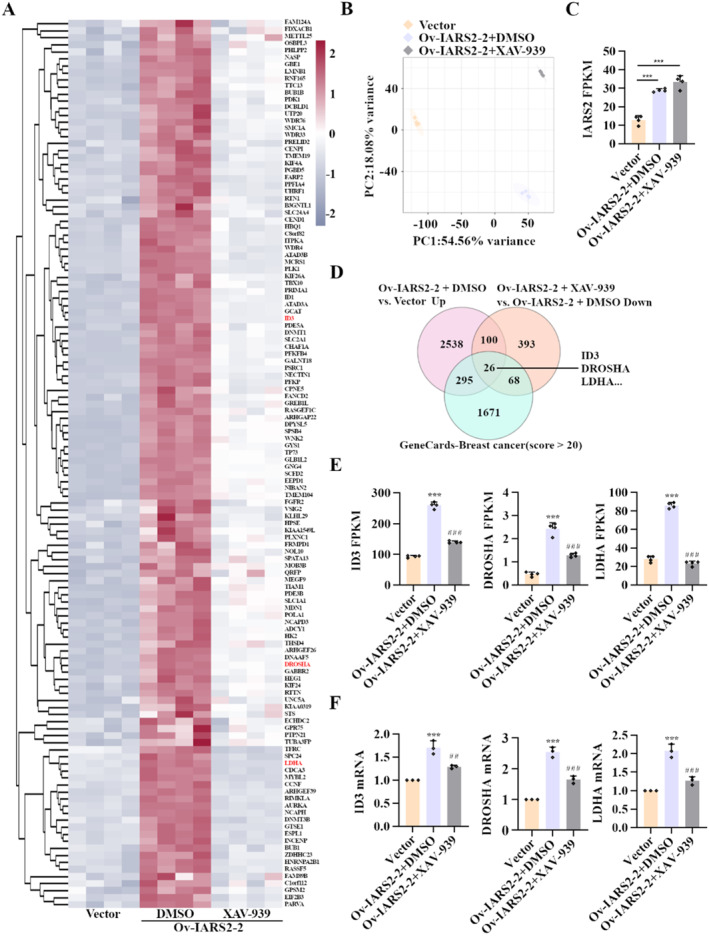
Transcriptome analyses were carried out on IARS2‐overexpressed MDA‐MB‐231 cells treated with XAV‐939. (A) The heatmap presented the 126 common DEGs upregulated in the Ov‐IARS2‐2 + DMSO group (compared with the vector group) and downregulated in the Ov‐IARS2‐2 + XAV‐939 group (compared with the Ov‐IARS2‐2 + DMSO group). (B) PCA plot for the vector, Ov‐IARS2‐2 + DMSO, and Ov‐IARS2‐2 + XAV‐939 group. (C) FPKM values of IARS2. (D) Venn diagram showing the intersection of three gene sets: (i) genes upregulated in Ov‐IARS2‐2 + DMSO versus vector group; (ii) genes downregulated in Ov‐IARS2‐2 + XAV‐939 versus Ov‐IARS2‐2 + DMSO; and (iii) breast cancer‐associated genes retrieved from GeneCards with a relevance score >20. The overlapping region contains 26 common genes, including LDHA, ID3, and DROSHA. (E) FPKM values of ID3, DROSHA, and LDHA. *n* = 4 per group. (F) Real‐time PCR measured the mRNA expression of ID3, DROSHA, and LDHA in the cells. *n* = 3 per group. ***p < 0.001 versus the vector group. ^##^ *p* < 0.01, ^###^ *p* < 0.001 versus the Ov‐IARS2‐2 + DMSO group. DEGs, differentially expressed genes; DMSO, dimethyl sulfoxide; DROSHA, Drosha ribonuclease III; FPKM, fragments per kilobase of transcript per million mapped reads; IARS2, isoleucyl‐tRNA synthetase 2; ID3, inhibitor of DNA binding 3; LDHA, lactate dehydrogenase A; Ov‐IARS2, IARS2‐overexpression plasmids; PCA, principal component analysis.

Based on mRNA‐seq analysis, upregulated DEGs in the Ov‐IARS2‐2 + DMSO group compared with the vector groups and downregulated DEGs in the Ov‐IARS2‐A + XAV‐939 group compared with the Ov‐IARS2‐2 + DMSO group (genes reversed by β‐catenin inhibition) were identified (|log_2_FC| > 0.8 and *p* < 0.05). The intersection of these two sets yielded 126 common DEGs. These DEGs were displayed in the heatmap (Figure 9A). The distribution of these samples was visualized by principal component analysis (Figure 9B). As expected, the fragments per kilobase of transcript per million mapped reads values of IARS2 were upregulated in the IARS2‐overexpressed MDA‐MB‐231 cells treated with or without XAV‐939 (Figure 9C). To prioritize breast cancer‐relevant candidates, we intersected these 126 genes with the GeneCards‐annotated “Breast cancer” genes and applied a score filter (>20) to enrich for genes with strong disease association, which generated 26 genes (Figure 9D). Among these, inhibitor of DNA binding 3 (ID3),^26^, ^27^ Drosha ribonuclease III (DROSHA),^28^ and lactate dehydrogenase A (LDHA)^29^, ^30^ were selected for further validation based on the literature evidence, which indicates that ID3, DROSHA, and LDHA are downstream effectors of β‐catenin and are capable of promoting breast cancer. Interestingly, IARS2 overexpression upregulated the levels of ID3, DROSHA, and LDHA (log_2_FC > 0.8 and *p* < 0.05), which were downregulated by XAV‐939 in MDA‐MB‐231 cells (log_2_FC < −0.8 and *p* < 0.05) (Figure 9E). Real‐time PCR was used to verify the results (Figure 9F). To determine whether these candidates were functionally required for the pro‐proliferative effect of IARS2, we performed siRNA‐mediated knockdown in IARS2‐overexpressing MDA‐MB‐231 cells. IARS2‐stimulated cell proliferation was attenuated by knockdown of LDHA, ID3, or DROSHA (Supporting Information S1: Figure S6A). These results demonstrated that LDHA, ID3, and DROSHA are necessary for the proliferative phenotype driven by IARS2, supporting their role as downstream effectors of the IARS2/β‐catenin axis. Given these analyses, IARS2 might regulate the β‐catenin pathway, thereby leading to the progression of breast cancer.

## 4 DISCUSSION

Upregulated IARS2 was identified in BRCA based on the UALCAN‐TCGA database and GSE45827 microarray data. The UALCAN‐TCGA and Kaplan–Meier plotter (gene chip) databases displayed that elevated IARS2 expression correlated with reduced survival rates in patients with breast cancer. These findings suggested a potentially significant role for IARS2 in breast cancer progression. In this study, we validated that IARS2 expression was higher in breast cancer tissues compared to adjacent nontumor tissues, as anticipated. Our subsequent results demonstrated that IARS2 stimulated breast cancer cell progression. Furthermore, IARS2 accelerated mammary tumor formation in vivo. Notably, β‐catenin accumulation was enhanced by IARS2 in the mice injected with MDA‐MB‐231 cells. Mechanistically, IARS2 knockdown facilitated β‐catenin phosphorylation, thereby promoting β‐catenin ubiquitin‐proteasome degradation, which inhibited β‐catenin nuclear translocation and repressed its transcriptional activation of downstream target genes in MDA‐MB‐231 cells. Additionally, the β‐catenin inhibitor XAV‐939 attenuated the malignant behaviors of IARS2‐overexpressed MDA‐MB‐231 cells. To investigate the involvement of β‐catenin signaling in the IARS2‐mediated effects, mRNA‐seq was performed, and the expression and function of β‐catenin‐associated factors were validated. The study revealed that silencing IARS2 might contribute to the treatment of breast cancer via blocking the β‐catenin pathway.

Accumulating evidence shows that ARSs are linked to tumor development,^6^ and abnormal expression of several ARSs has been identified in breast cancer. Arginyl‐tRNA synthetase (RARS) is upregulated in MCF7 breast cancer cells.^31^ Lysyl‐tRNA synthetase is highly expressed in the tumors of patients with breast cancer.^32^ Compared with adjacent normal tissues, glutamyl‐prolyl‐tRNA synthetase (EPRS) transcript levels are elevated in ER+ breast cancer tissues. EPRS exerts a critical role in the proliferation of ER+ breast cancer cells.^33^ Researchers have discovered that alanyl‐tRNA synthetase, histidyl‐tRNA synthetase, RARS, and tryptophanyl‐tRNA synthetase have a significant correlation with increased breast cancer risk in a Chinese population.^34^ However, so far, there has been no report that IARS2 affects breast cancer. Herein, we initially identified high expression of IARS2 in breast cancer, correlated with poor prognosis, through analysis of the public databases. Subsequently, validation using clinical samples revealed that IARS2 expression was upregulated in breast cancer tissues compared to adjacent nontumor tissues. Further IHC analysis displayed high IARS2 levels in the tumor tissues. These findings suggested that IARS2 might be carcinogenic in breast cancer. In fact, IARS2 has been reported in various cancers. For instance, IARS2 knockdown reduces cell proliferation and stimulates cell apoptosis in non‐small‐cell lung cancer,^9^ gastric cancer,^11^ and osteosarcoma.^15^ Consistently, breast cancer cell proliferation was constricted, and cell apoptosis was accelerated by silencing IARS2. Besides, knockdown of IARS2 led to breast cancer cell cycle arrest. In vivo, IARS2 deficiency contributed to the suppression of breast cancer cell tumorigenicity. IARS2 overexpression caused the opposite effects.

It is widely known that aberrant activation of β‐catenin signaling is related to malignant phenotype progression, poor prognosis, and an increase in cancer‐linked mortality.^18^ Multiple studies have uncovered that β‐catenin participates in the progression of malignant behaviors in breast cancer.^35^ The receptor for activated C kinase 1 advanced breast cancer progression by impeding the binding between ubiquitinated β‐catenin and 26S proteasome non‐ATPase regulatory subunit 2 to increase β‐catenin stability.^36^ Prospero‐related homeobox 1 activates the Wnt/β‐catenin pathway, thereby facilitating breast cancer invasion and metastasis.^37^ The exploration of β‐catenin inhibitors is of great significance for the treatment of breast cancer.

IARS2 knockdown suppresses β‐catenin expression in human umbilical vein endothelial cells.^18^ IARS2 is capable of stabilizing β‐catenin, thereby enhancing cell proliferation and metastasis in pancreatic ductal adenocarcinoma.^19^ Given these reported results, IARS2 might affect breast cancer through regulating β‐catenin signaling. In this study, we investigated the effects of IARS2 on β‐catenin stability in breast cancer cells. The results demonstrated that silencing IARS2 promoted β‐catenin ubiquitination and degradation and obstructed the entry of β‐catenin into the nucleus in MDA‐MB‐231 cells. XAV‐939, as a β‐catenin inhibitor, abrogated the effects of IARS2 overexpression in the cells. Consequently, it was suggested that IARS2 knockdown might inhibit the transcriptional activation of β‐catenin‐related downstream target genes to exert anticancer effects in breast cancer.

Further, transcriptome sequencing was performed to investigate the IARS2‐regulated downstream target genes related to β‐catenin. Based on mRNA‐seq results, IARS2 was upregulated in the Ov‐IARS2‐2‐transfected MDA‐MB‐231 cells treated with DMSO and XAV‐939 compared with the cells transfected with vector as anticipated. To our knowledge, there is no research reporting the effects of XAV‐939 on IARS2 expression. The mRNA‐seq results showed that IARS2 expression has no significant changes after XAV‐939 treatment in the IARS2‐overexpressed cells. Importantly, the expression and function of breast cancer‐linked ID3, DROSHA, and LDHA were verified in the cells. For ID3, angiopoietin‐like 4 leads to β‐catenin translocation to the nucleus and transcriptionally upregulates ID3 and thus diminishes scar collagen expression in fibroblasts.^26^ β‐Catenin/ID3 affects the polarization of M2 macrophages, thereby promoting lung cancer metastasis.^27^ Notably, ID3 stimulates human MCF‐7 breast cancer cell proliferation and invasive growth.^38^ A study has revealed that DROSHA interacts with β‐catenin to accelerate STC1 transcription, which contributes to breast cancer stem‐like cell properties.^28^ In addition, the binding of β‐catenin to the LDHA promoter has been proved in lung cancer cells.^39^ The literature has suggested that β‐catenin targets c‐Myc to activate LDHA and hexokinase 2, exerting the effects of tumor promotion in colorectal cancer^29^ and cervical cancer.^30^ Activating LDHA has been reported to facilitate the malignant process of breast cancer.^40^ In this work, we discovered that overexpressing IARS2 caused the ascendance of the expression of ID3, DROSHA, and LDHA, whereas XAV‐939 downregulated the expression of these factors in the breast cancer cells according to mRNA‐seq results. Silencing ID3, DROSHA, and LDHA was capable of reversing IARS2‐promoted cell proliferation in MDA‐MB‐231 cells. Moreover, β‐catenin downstream classic target genes, such as c‐Myc, cyclin D1, and Axin‐2, were inhibited by IARS2 knockdown and augmented by IARS2 overexpression in the tumor tissues. Taken together, these results indicated that IARS2, by stabilizing β‐catenin and enhancing its transcriptional activity, may orchestrate a transcriptional program involving β‐catenin targets (ID3, DROSHA, LDHA, c‐Myc, cyclin D1, and Axin‐2) to promote breast cancer progression.

It is important to acknowledge the limitations of this study. First, IHC analysis of IARS2 protein expression was performed only on 56 breast cancer tissues without matched normal counterparts due to the limited availability of normal tissue sections in the paraffin‐embedded samples. Future studies incorporating matched normal breast tissues will be necessary to fully validate the protein‐level expression of IARS2 in breast cancer. Second, the functional rescue experiments demonstrating that IARS2 exerted its oncogenic effects through β‐catenin were performed only in MDA‐MB‐231 cells. Future studies employing additional breast cancer cells will further substantiate the generalizability of our findings. Third, although we have demonstrated that IARS2 regulated β‐catenin expression and downstream target genes (c‐Myc, cyclin D1, and Axin‐2) in vivo, more direct mechanistic evidence, such as β‐catenin ubiquitination levels or transcriptional activity in tumor tissues, was not assessed due to experimental constraints. Future studies employing additional in vivo models and techniques will further elucidate the precise molecular mechanisms by which IARS2 modulates β‐catenin signaling. Fourth, the precise mechanisms by which IARS2 controls the transcription or activity of ID3, DROSHA, and LDHA remain to be fully elucidated. It should be emphasized that these three genes are not the only or essential downstream effectors of the IARS2/β‐catenin axis in breast cancer. There may be many other factors involved in breast cancer regulation associated with β‐catenin. This warrants further investigation.

In conclusion, elevated IARS2 expression was confirmed in breast cancer tissues compared to adjacent nontumor tissues. IARS2 stimulated breast cancer cell progression. Furthermore, IARS2 enhanced tumor growth in mice injected with breast cancer cells. Mechanistically, IARS2 upregulated β‐catenin expression in the murine xenograft models. Knockdown of IARS2 promoted ubiquitination‐mediated degradation of β‐catenin in breast cancer cells. IARS2 might serve as a tumor promoter in breast cancer by activating the β‐catenin signaling pathway.

## AUTHOR CONTRIBUTIONS

**Xi Yang**: Writing—original draft; software; methodology; investigation; conceptualization. **Ya Wang**: Writing—original draft; software; methodology; investigation. **Yanjiao Yi**: Software; investigation; formal analysis; data curation. **Yang Yang**: Investigation; data curation. **Hongjiang Wang**: Writing—review and editing; methodology; conceptualization.

## CONFLICT OF INTEREST STATEMENT

The authors declare no conflicts of interest.

## ETHICS STATEMENT

This study was approved by the Experimental Animal Ethical Committee of Dalian Medical University (AEE24319). All patients provided informed consent. The study was performed following the Declaration of Helsinki.

## Supporting information

Supporting Information S1

## Data Availability

All data generated or analyzed during the study are available from the corresponding author upon reasonable request.
