# Supplementary material for: Unveiling isoleucyl‐tRNA synthetase 2 as a novel driver of breast cancer via β‐catenin pathway activation
Source: J Cell Commun Signal. 2026 Jul 12;20(3):e70094. doi: 10.1002/ccs3.70094 (PMC13356825; doi:10.1002/ccs3.70094)
Supplement: Supplementary file 1 — Supporting Information S1 [file CCS3-20-e70094-s001.docx]

**Supporting Information**

**MATERIALS AND METHODS**

**Cell culture**

MDA-MB-231 cells were derived from the breast cancer tissue of a 51-year-old woman (RRID: CVCL_0062). BT-20 cells were obtained from the breast cancer tissue of a 74-year-old woman (RRID: CVCL_0178). The cell lines were authenticated for the described experiments and have not been previously reported as misidentified or contaminated. MDA-MB-231 and BT-20 cells were determined to be free of mycoplasma contamination. MDA-MB-231 cells were cultured in MEM medium (Solarbio, Beijing, China) and BT-20 cells were maintained in Leibovitz's L-15 medium (Procell, Hubei, China) at 37°C in an incubator with 5% CO_2_. Both of the mediums contained 10% fetal bovine serum (Tianhang, Zhejiang, China) and 1% penicillin and streptomycin.

**Cell treatment**

The cells treated with 100 µg/mL CHX were harvested at 0, 4, 8, 12 and 24 h for β-catenin protein measurement. Additionally, the MDA-MB-231 cells were subjected to 10 µM MG132 treatment for 4 h, and then the ubiquitination of β-catenin was detected. To investigate whether IARS2 affects the phenotype of breast cancer cells by regulating β-catenin signaling, the MDA-MB-231 cells transfected with Ov-IARS2-2 were treated with 20 µM β-catenin inhibitor XAV-939 for 24 h.

For functional validation of downstream effector factors of β-catenin, the following siRNA sequences (sense strand) were used: Inhibitor of DNA binding 3 siRNA (si-ID3: 5’-CGGAACUUGUCAUCUCCAATT-3’), Drosha ribonuclease III siRNA (si-DROSHA: 5’-GGGAGAUUCUACAGUGGUUTT-3’) and Lactate dehydrogenase A siRNA (si-LDHA: 5’-CUGGCAAAGACUAUAAUGUTT-3’). A non‑targeting negative control siRNA (si‑NC) was used as control. IARS2‑overexpressed stable MDA‑MB‑231 cells (Ov-IARS2-2) or empty vector cells (Vector) were transfected with si-ID3, si-DROSHA, si-LDHA or si-NC. The cells were harvested at 48 h post‑transfection for subsequent assays.

**Xenograft mouse model**

Before tumor cell inoculation, six-week-old female BALB/c nude mice were randomized into different groups, including sh-NC, sh-IARS2-3, Vector and Ov-IARS2-2. The MDA-MB-231 cells stably infected with sh-IARS2-3 or Ov-IARS2-2 were injected subcutaneously (1×10^7^ cells) into the right axilla of the mice (n=6 per group). The mice injected with the MDA-MB-231 cells transfected with sh-NC or Vector were considered as the control. One week later, the width and length of tumor were determined every 2 days. Tumor volume was calculated by the formula: volume = 0.5×length×width^2^. Three weeks later, the mice were sacrificed, and tumors were removed and weighed.

**IHC**

The human breast cancer tissues as well as mouse tumors were fixed in 4% paraformaldehyde. The fixed tissues were dehydrated and embedded in paraffin. Next, the samples were mounted on an automatic microtome (Leica, German) and cut into 5μm sections. The slices were deparaffinized with xylene and hydrated with gradient alcohol. The sections were put into a microwave oven to repair the antigen for 10 min and subsequently cooled at room temperature. The activity of endogenous peroxidase was eliminated using 3% H_2_O_2_. The tissues were then blocked with 1% bovine serum albumin for 15 min. The tissues were incubated overnight at 4°C with the primary antibody anti-IARS2 (Proteintech, Hubei, China; 1:100) and anti-β-catenin (Proteintech, Hubei, China; 1:100). Secondary antibody HRP-labeled goat anti-rabbit IgG (ThermoFisher, USA; 1:500) was added and incubated with the slices at 37°C for 60 min. The diaminobenzene (DAB) and hematoxylin were applied for staining. At last, the samples were dehydrated with gradient alcohol, mounted, and photographed under a microscope (Olympus, Japan) at 400× magnification.

**Cell** **proliferation**

Briefly, the cells were seeded in 96-well plates (5×10^3^ cells/well). After culturing for 0, 24, 48 and 72 h, 10 µL CCK-8 (Biosharp, Anhui, China) was supplemented to the cells. Afterwards, the cells were incubated at 37°C in an incubator with 5% CO_2_ for 2 h. The absorption at 450 nm was measured by a microplate reader (BioTek, USA).

**Cell cycle and apoptosis**

For cell cycle determination, the cells were harvested after centrifugation at 150 g for 5 min. Then, the cells were washed with phosphate buffered saline (PBS), removed the supernatant, fixed with 70% alcohol at 4℃ overnight. After that, 500 µL PI/RNase A staining solution (RNase A: PI=1:9) incubated the cells in the dark for 30 min. At last, NovoCyte Flow Cytometer (Agilent, USA) was used to analyze the cell cycle.

For cell apoptosis examination, the cells were centrifuged and the supernatant was discarded. The cells were resuspended with 500 µL binding buffer. Subsequently, 5 µL propidium iodide (PI) were blended in the cells following mixing with 5 µL AnnexinV-FITC. The cells were incubated in the dark for 10 min at room temperature. The number of apoptotic cells was measured by NovoCyte Flow Cytometer (Agilent, USA). Besides, we detected apoptosis-related caspase-9 and caspase-3 activity in the transfected MDA-MB-231 and BT-20 cells based on the manufacturer’s instructions using the corresponding kits (Solarbio, Beijing, China).

**Cell migration**

Cell migration was assessed using a wound healing assay. Before scratching, the medium was replaced with serum‑free medium containing mitomycin C (Sigma, USA) and incubated for 1 h to inhibit cell proliferation. A sterile 200 µL pipette tip was then used to create a linear scratch. The detached cells were gently washed away with serum‑free medium, and images of the scratched area were captured under a phase‑contrast microscope (100× magnification) at 0 and 24 h. The wound closure distance was quantified.

**Cell invasion**

Matrigel (Corning, USA) ‑coated Transwell chambers (LABSELECT, USA) were used. The cell suspension was seeded into the upper chamber, and the lower chamber was added with culture medium contained 10% FBS. After 24 h, the cells were fixed with 4% paraformaldehyde (Aladdin, Shanghai, China) and stained with crystal violet (Amresco, USA). Five random fields per sample were counted in the cells on the lower surface under a phase‑contrast microscope (200× magnification), and the average number of invaded cells was calculated.

**Immunofluorescence**

The stable IARS2-knockdown breast cancer cells were fixed in 4% paraformaldehyde (Sinopharm, Shanghai, China) for 15 min. After being washed with PBS, the cells were incubated with 0.1% tritonX-100 (Beyotime, Shanghai, China) for 30 min at room temperature. The cells were blocked with 1% bovine serum albumin (BSA) for 15 min and incubated with anti-β-catenin (Proteintech, Hubei, China; 1:100) at 4℃ overnight. Cy3-marked goat anti-rabbit IgG (Abcam, Shanghai, China; 1:200) was added to the cells in the dark and incubated for 60 min. DAPI (Aladdin, Shanghai, China) was used to stain the nuclei. Images were observed under a fluorescence microscope (Olympus, Japan).

**Dual luciferase reporter assay**

Luciferase activities were determined by the Dual Luciferase Reporter Gene Assay Kit (Biosharp, Anhui, China) according to the manufacturer’s protocol. The breast cancer cells were transfected with β-catenin responsive TOPflash and pRL-TK luciferase reporter (TOPflash: pRL-TK=10:1) using lipofectamine 3000. After transfection for 48 h, ratios of Firefly and Renilla luciferase activity values were calculated to reflect the activity of β-catenin.

**Real-time PCR**

TRIpure lysis solution (BioTeke, Beijing) was used to isolate the total RNA from the breast cancer tissues or cells. The cDNA was synthesized using All-in-One First-Strand SuperMix (Magen, Guangzhou, China). The real-time PCR was performed using 2×Fast Taq plus PCR Master Mix (Biosharp, Anhui, China), SYBR Green (Solarbio, Beijing, China) and the following primers: IARS2 forward: 5’-AGTTGTTCGGCTGTTAC-3’, IARS2 reverse: 5’-CACTCTTGGGCTCTTTA-3’; c-Myc forward: 5’-CACCCTTCTCCCTTCGG-3’, c-Myc reverse: 5’-CAGTCCTGGATGATGATGTTT-3’; Cyclin D1 forward: 5’-GATGCCAACCTCCTCAACGA-3’, Cyclin D1 reverse: 5’-GGAAGCGGTCCAGGTAGTTC-3’; Axin-2 forward: 5’-TGACGGACAGCAGTGTAGATG-3’, Axin-2 reverse: 5’-GTTCTCGGGAAATGAGGTAG-3’; β-actin forward: 5’-AAATCTGGCACCACACCTTC-3’, β-actin reverse: 5’-GGGGTGTTGAAGGTCTCAAA-3’. In addition, we also validated the expression of β-catenin pathway-related factors in mRNA-seq results using real-time PCR, with the following primers: Inhibitor of DNA binding 3 (ID3) forward: 5’-GCCTGCGGGAACTGGTA-3’, ID3 reverse: 5’-CTGGATGGGAAGGTGGG-3’; Drosha ribonuclease III (DROSHA) forward: 5’-ATGCCCGAACCTACACT-3’, DROSHA reverse: 5’-CATCCATTGCTGCTCCC-3’; Lactate dehydrogenase A (LDHA) forward: 5’-GTGCCTGTATGGAGTGG-3’, LDHA reverse: 5’-TGTAGCCTTTGAGTTTGAT-3’; β-actin forward: 5’-TCAGGGTGAGGATGCCTCTC-3’, β-actin reverse: 5’-CTCGTCGTCGACAACGGCT-3’. β-actin was selected as an internal reference gene. Relative quantitative expression of the genes was calculated by the 2^−ΔΔCt^ method.

**Co-immunoprecipitation (Co-IP)**

Briefly, the cells were lysed using native lysis buffer (Solarbio, Beijing, China). The cell lysates were centrifuged at 10000 g for 5 min at 4 ℃ by a high-speed freezing centrifuge (Xiangyi, Hunan, China), and then the supernatants were collected for the following experiments. The proteins were quantified by the BCA protein assay kit (Solarbio, Beijing, China). β-catenin antibody (Proteintech, Hubei, China) was immobilized into AminoLink coupling resin by incubating on a rotator at room temperature for 120 min. The lysates were added into the washed resin and the mixtures were oscillated for 2 h. The next day, 200 µL IP lysis/wash buffer was added. The samples were centrifuged after each wash. Subsequently, the samples were eluted with elution buffer. 9% sodium dodecyl sulfate polyacrylamide gel electrophoresis (SDS-PAGE) was used to separate the proteins in the samples. The immunoprecipitated proteins were analyzed by western blot as described below. The primary antibody is ubiquitin antibody (Proteintech, Hubei, China; 1:5000), and the secondary antibody is HRP-labeled goat anti-rabbit IgG (Solarbio, Beijing, China; 1:3000). Reserved whole cell lysates were used as positive controls.

**Western blot**

The breast cancer cells and the tumors were lysed by RIPA buffer (Solarbio, Beijing, China) to obtain proteins, which were quantified by the BCA protein assay kit (Solarbio, Beijing, China). The proteins were separated by 8% and 9% SDS-PAGE and transferred onto polyvinylidene difluoride (PVDF) membranes (Millipore, USA). The membranes were blocked with blocking buffer (Solarbio, Beijing, China) for 1 h. Next, the samples were incubated with anti-rabbit primary antibodies against IARS2 (Proteintech, Hubei, China; 1:2000), cyclin D1 (Zenbio, Sichuan, China; 1:1000), p21 (Zenbio, Sichuan, China; 1:1000), Bax (Zenbio, Sichuan, China; 1:500), Bcl-2 (Zenbio, Sichuan, China; 1:500), β-catenin (Proteintech, Hubei, China; 1:5000), p-β-catenin Ser33/37/Thr41 (Affinity Biosciences, Changzhou, China; 1:1000) or anti-mouse β-actin (Santa Cruz, USA; 1:1000) at 4℃ overnight. β-actin was used as a protein-loading control. The members were washed by Tris buffered saline (TBST) with Tween-20 and incubated with secondary antibodies HRP-labeled goat anti-rabbit IgG (Solarbio, Beijing, China; 1:3000) or goat anti-mouse IgG (Solarbio, Beijing, China; 1:5000) at room temperature for 1 h. The immunoblotted protein signals were monitored using an ECL western blotting substrate (Solarbio, Beijing, China).

**Supplementary Figures**


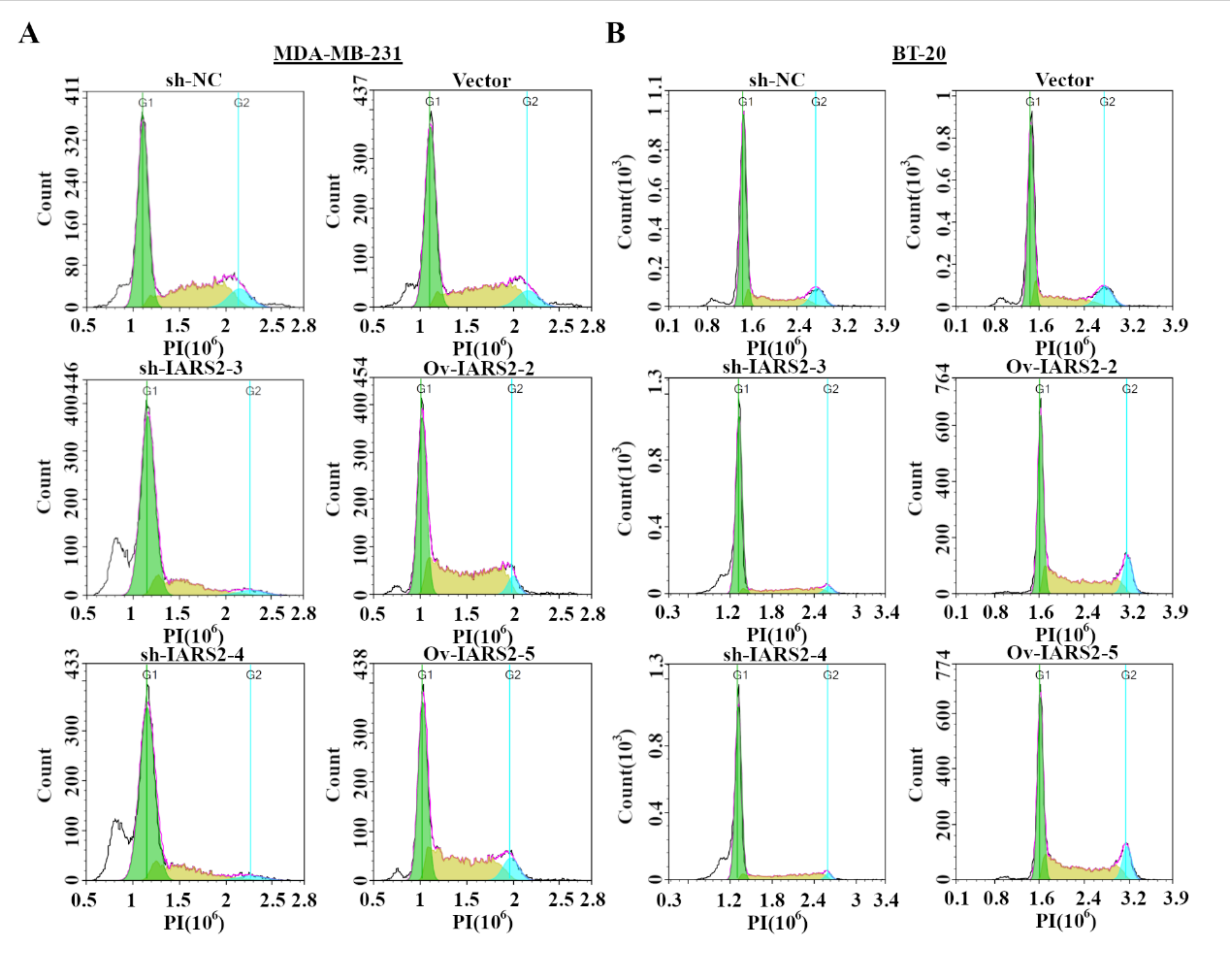


**FIGURE S1.** Flow cytometry analyzed cell cycle progression in the breast cancer cells after knocking down or overexpressing IARS2. (A) MDA-MB-231 cells. (B) BT-20 cells. n=3 per group.


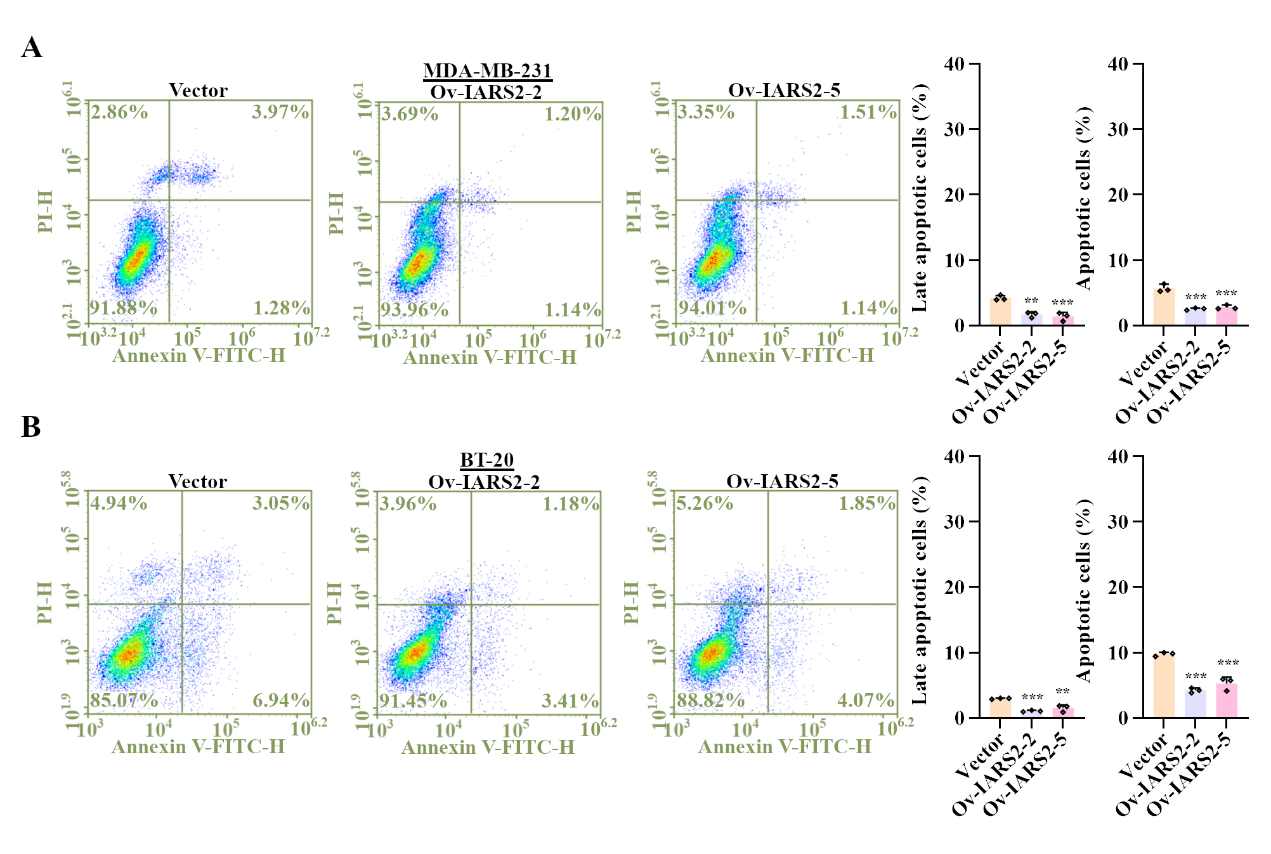


**FIGURE S2.** IARS2 blocked cell apoptosis in breast cancer cells. (A, B) Cell apoptosis was determined by flow cytometry in the IARS2-overexpressed MDA-MB-231 cells and BT-20 cells. The rates of late apoptotic cells and apoptotic cells were calculated and analyzed. n=3 per group. ***P* < 0.01, ****P* < 0.001.

**
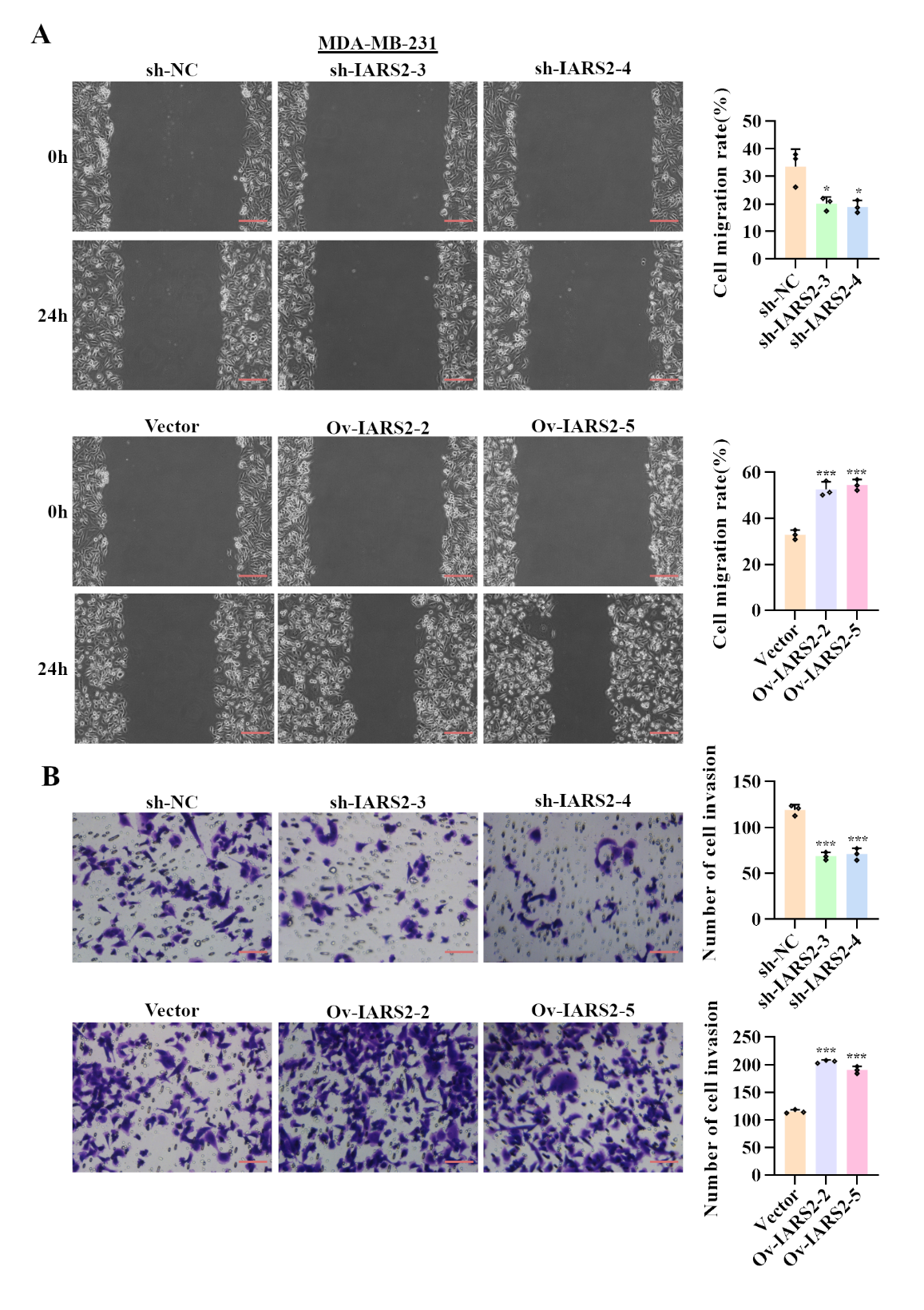
**

**FIGURE S3.** IARS2 regulated MDA‑MB‑231 cell migration and invasion. (A) Representative images of wound healing assays at 0 and 24 h in MDA‑MB‑231 cells. Scale bar is 200 μm. Cell migration rates were calculated. (B) Representative images of Transwell invasion assays. Scale bar is 100 μm. The number of invaded cells were quantified. n=3 per group. **P* < 0.05, ****P* < 0.001.

**
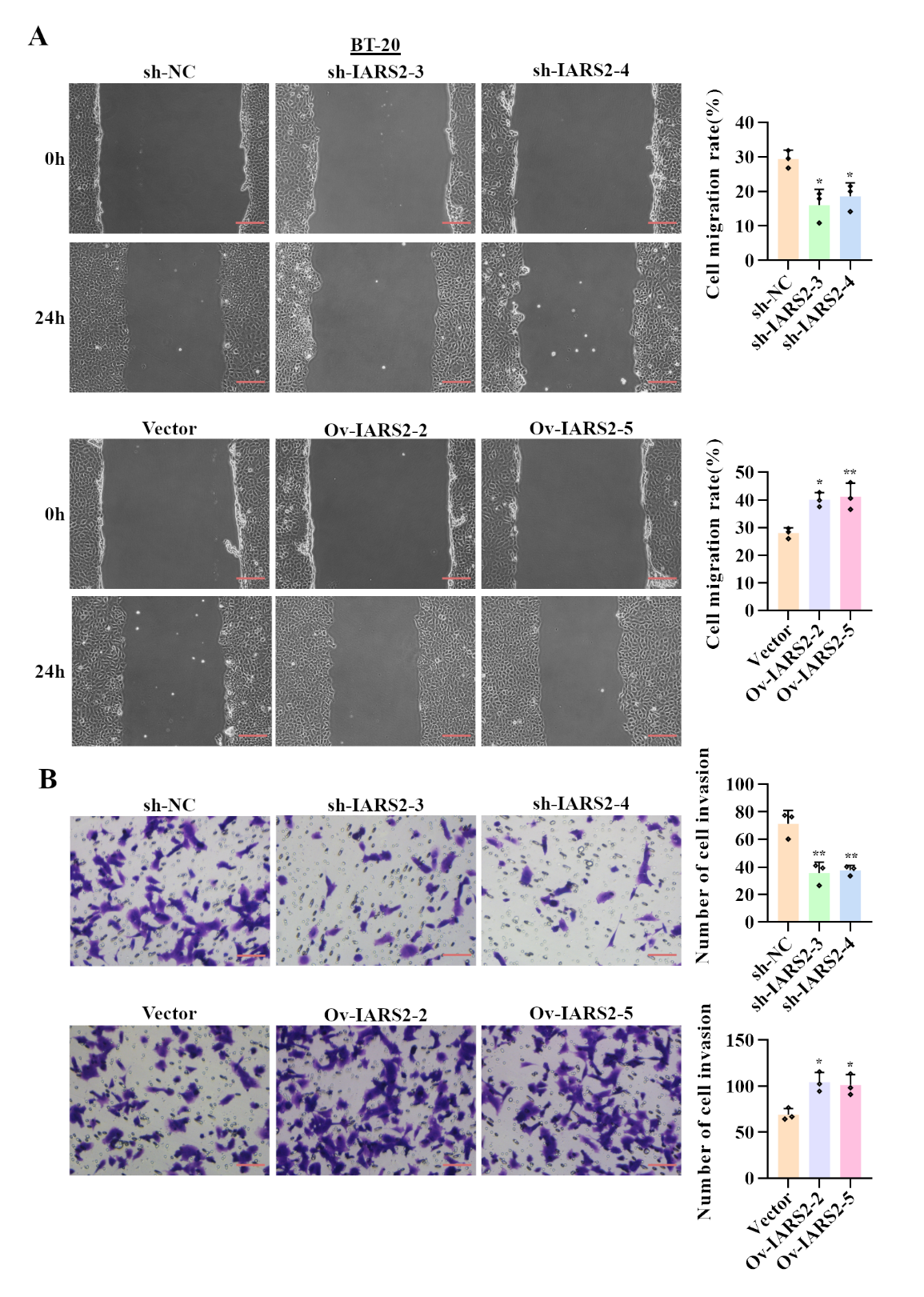
**

**FIGURE S4.** IARS2 modulated BT-20 cell migration and invasion. (A) Representative images of wound healing assays at 0 and 24 h in BT-20 cells. Scale bar is 200 μm. Cell migration rates were calculated. (B) Representative images of Transwell invasion assays. Scale bar is 100 μm. The number of invaded cells were quantified. n=3 per group. **P* < 0.05, ***P* < 0.01.

**
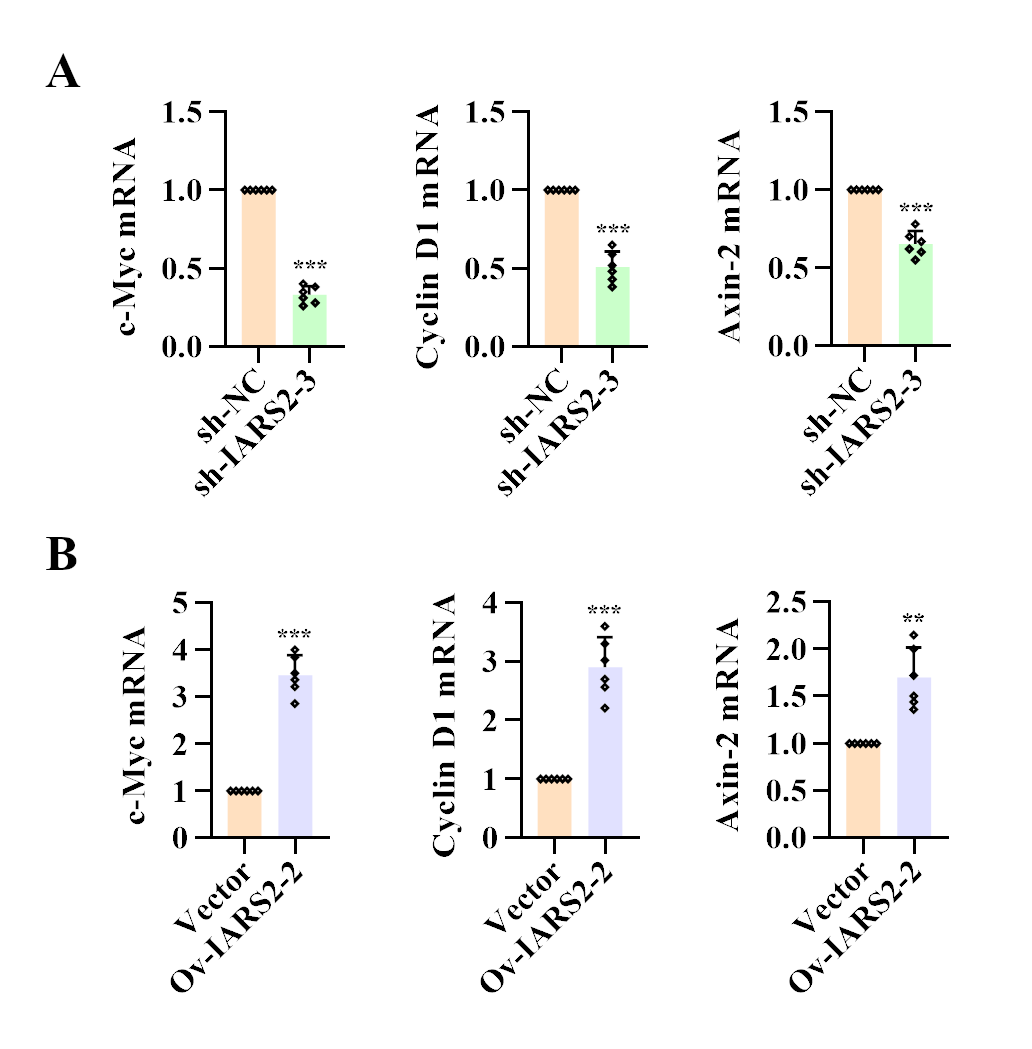
**

**FIGURE S5.** IARS2 regulated the expression of β-catenin-targeted c-Myc, Cyclin D1 and Axin-2 in tumor tissues of xenograft mouse model. (A, B) c-Myc, Cyclin D1 and Axin-2 mRNA levels were assessed by real-time PCR in the tumors with sh-IARS2-3 and Ov-IARS2-2. n=6 per group. ***P* < 0.01, ****P* < 0.001.

**
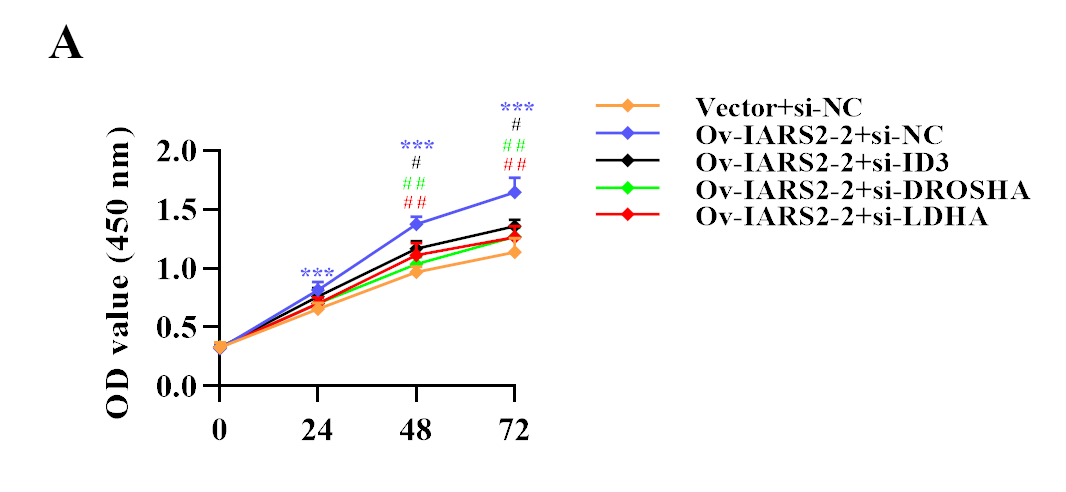
**

**FIGURE S6.** Functional rescue of IARS2‑induced proliferation by silencing β‑catenin downstream effector genes in breast cancer cells. (A) IARS2-overexpressed MDA‑MB‑231 cells were transfected with siRNAs targeting LDHA, ID3, DROSHA, or negative control siRNA (si‑NC). Cell proliferation was assessed by CCK‑8 assay. n=3 per group. ****P* < 0.001 vs. Vector group. ^#^*P* < 0.05, ^##^*P* < 0.01 vs. Ov-IARS2-2+DMSO group.
